# The role of melatonin as an adjuvant in the treatment of COVID-19: A systematic review

**DOI:** 10.1016/j.heliyon.2022.e10906

**Published:** 2022-10-07

**Authors:** Arezoo Faridzadeh, Arefeh Tabashiri, Hamid Heidarian Miri, Mahmoud Mahmoudi

**Affiliations:** a Immunology Research Center, Mashhad University of Medical Sciences, Mashhad, Iran; b Department of Immunology and Allergy, School of Medicine, Mashhad University of Medical Sciences, Mashhad, Iran; c Student Research Committee, School of Medicine, Shahid Beheshti University of Medical Sciences, Tehran, Iran; d Social Determinants of Health Research Center, Mashhad University of Medical Sciences, Mashhad, Iran

**Keywords:** COVID-19, Systematic review, Melatonin

## Abstract

**Introduction:**

Since November 2019, the world has been grappling with the rapid spread of the Coronavirus disease 2019 (COVID-19). In response to this major health crisis, the first vaccination rollout was launched in December 2020. However, even fully vaccinated individuals are not completely immune to infection, albeit with less severe symptoms. Melatonin is known as an anti-oxidant, anti-inflammatory, and immunomodulatory agent whose anti-viral properties, cost-effectiveness, and relatively few side effects make it a potential adjuvant in the treatment of COVID-19. This systematic review aims to summarize the clinical studies on the effects of melatonin on COVID-19 patients.

**Methods:**

The search of articles was carried out in the Web of Science, PubMed/MEDLINE, Cochrane library, and Scopus databases up to January 2022.

**Results:**

Ten articles were included in our study. It seems melatonin can decrease inflammatory markers, inflammatory cytokines, and the expression of some genes, including the signal transducer and activator of transcription (STAT)4, STAT6, T-box expressed in T cell (T-bet), GATA binding protein 3 (GATA3), apoptosis-associated speck-like protein containing a caspase recruitment domain (ASC), and caspase-1 (CASP1). In addition, melatonin appears to alleviate some clinical signs and symptoms and accelerate recovery. The use of melatonin in severe cases reduces thrombosis, sepsis, and mortality rate.

**Conclusion:**

This systematic review highlights the probable role of melatonin as a potential adjuvant in the treatment of COVID-19 after about two weeks of consumption. However, further high-quality randomized clinical trials are required.

## Highlights

• Melatonin may be an effective supplemental treatment for COVID-19.
• Melatonin can reduce inflammation, clinical signs and symptoms, and recovery time.
• Melatonin can reduce thrombosis, sepsis, and mortality rate.

## 1 Introduction

Since the emergence of the first case of coronavirus disease 2019 (COVID-19) in late December 2019 in Wuhan, the world has been in a drawn-out battle against a pandemic that continues to this day. According to the World Health Organization (WHO), as of 10 August, more than 583 million confirmed cases and more than 6.4 million deaths have been reported worldwide (WHO Coronavirus).

Although the COVID-19 vaccination started in December 2020, only 51.2% of the world population has been fully or partially vaccinated, and this number is much lower in low-income countries (Coronavirus Vaccinations; Mathieu et al., 2021). Besides, even the fully vaccinated population is still susceptible to contracting the virus, albeit with less severe symptoms (CDC).

Melatonin is an endocrine molecule secreted by the pineal gland. It is also synthesized in mitochondria throughout the body. This tissue melatonin is manyfold greater in quantity than melatonin from the pineal gland (Melhuish Beaupre et al., 2021; Suofu et al., 2017). In addition to its well-known role in sleep and circadian rhythm regulation, it is also known as an anti-inflammatory, anti-oxidant, and immunomodulatory agent (Vlachou et al., 2021). Besides these properties, it is a cost-effective anti-viral with minor side effects, which makes it a potential adjuvant in the treatment of COVID-19 (Bahrampour Juybari et al., 2020).

The benefits of melatonin in the treatment of viral infections can be attributed to its properties as an immune function stimulator, an anti-oxidant enzyme inducer, a free radical scavenger, and an apoptosis regulator (Boga et al., 2012). Some studies on animals also support the anti-viral effects of melatonin against certain infections such as those caused by encephalomyocarditis virus, Semliki Forest virus, West Nile virus, Venezuelan equine encephalitis virus, and Aleutian mink disease virus (Ben-Nathan et al., 1995; Bonilla et al., 1997; Ellis, 1996).

Several studies have pointed to the use of melatonin in treating COVID-19 patients and have described the possible mechanisms involved (Reiter et al., 2020a; Reiter et al., 2020b; Shneider et al., 2020; Tan and Hardeland, 2020; Zhang et al., 2020a). Many studies have highlighted the link between COVID-19 and cytokine storm. According to the available clinical data, the severity of COVID-19 and the resulting death are associated with the cytokine storm (Hu et al., 2021). Cytokine storm causes excessive production of reactive oxygen species (ROS), leading to clinical signs such as reduced oxygen saturation. Besides, the cytokine storm leads to the over-activation of neutrophils, which produce myeloperoxidase (MPO). Melatonin is both a potent ROS scavenger and a potent MPO inhibitor. This is one of the possible mechanisms by which melatonin can act as a therapeutic agent against COVID-19 (Camp et al., 2021). Although it seems that melatonin can help to alleviate the symptoms and lessen the inflammatory response in COVID-19 patients, few clinical studies are available.

In this paper, we systematically review the clinical studies on melatonin's effects in the treatment of COVID-19 patients. There have been three previously-published systematic reviews on the same topic; however, they contained a very small number of trial studies, and the main focus of one study was molecular bases and animal models (Corrao et al., 2021; Gholizadeh et al., 2021; Lan et al., 2022).

## 2 Methods

This systematic literature review was carried out according to the guidelines set by the Preferred Reporting Items for Systematic Reviews and Meta-Analyses (PRISMA) statement (Moher et al., 2009; PRISMA 2020). The systematic review has been registered on PROSPERO under ID number CRD42021284059.

### 2.1 Eligibility criteria

We used the PICO (Population, Intervention, Comparison, and Outcomes) framework: Population: adults older than 18 years of age with COVID-19 (diagnosed by Computed Tomography (CT) scan or nasopharyngeal swab Reverse Transcription Polymerase Chain Reaction (RT-PCR)); Intervention: melatonin; Comparison: comorbidities, gender, severity, melatonin dosage, and duration; Outcomes: change in inflammatory markers, clinical signs and symptoms from baseline to the last available follow-up.

### 2.2 Data source and search strategy

The search of articles was carried out in the Web of Science, PubMed/MEDLINE, Cochrane library, and Scopus databases from 2 October 2021 until the time of the article's submission. The newest published article found by the manual search was added to this review, without language restrictions, using the keywords (melatonin) AND (“COVID-19” OR “COVID 19” OR “SARS-COV-2” OR ″2019-nCoV" OR "Coronavirus Disease-19″ OR “Coronavirus Disease 19” OR “2019 Novel Coronavirus” OR “2019 nCoV” OR “Coronavirus Disease, 2019”). The last search date was January 28, 2022.

### 2.3 Study selection

**Figure 1:**
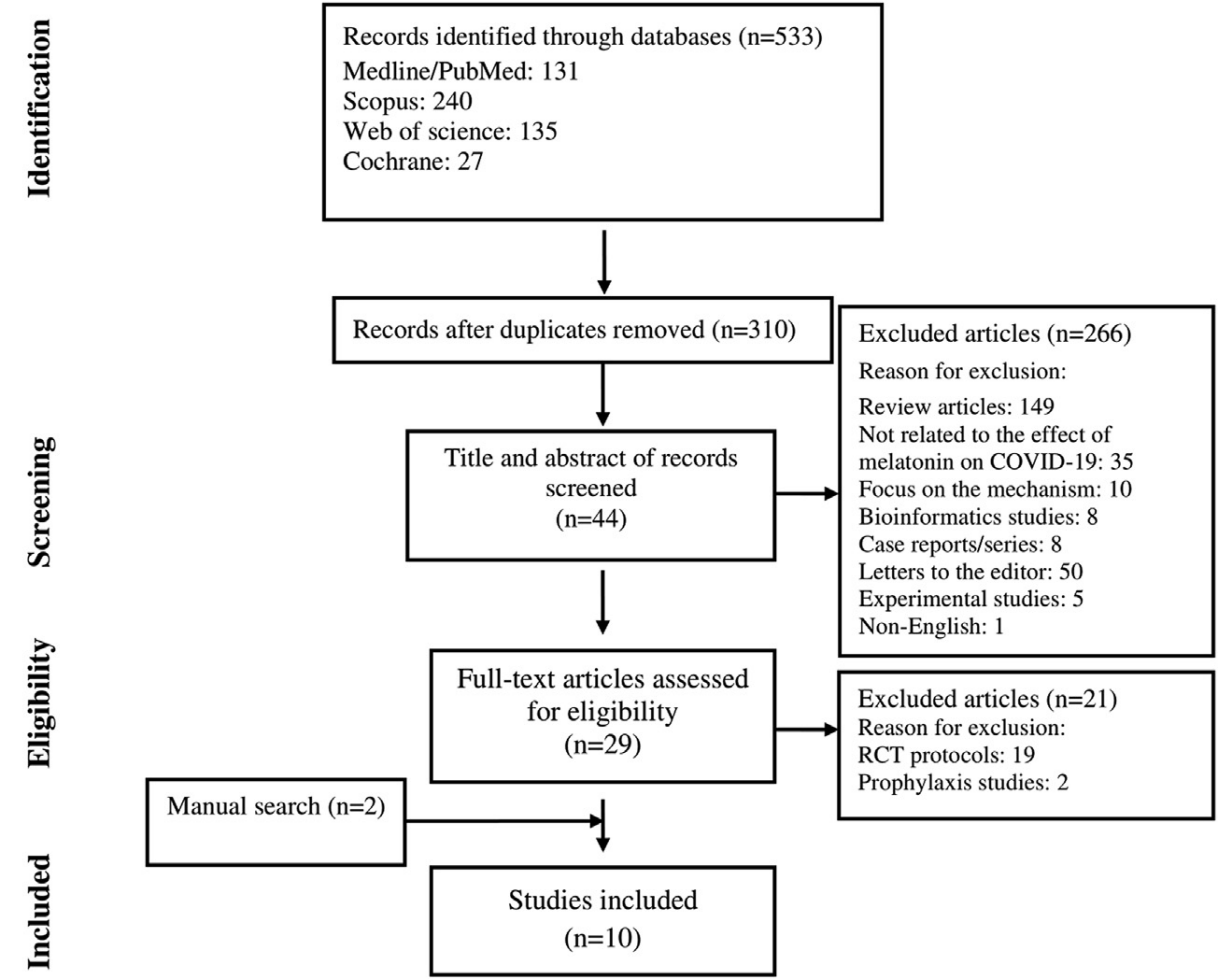
Flow chart of study selection for inclusion in the systematic review.

Two reviewers independently screened and identified articles by assessing their titles and abstracts. After removing the duplicates, if the title or abstract clearly indicated that a study was not relevant, it was excluded from further assessment. Such studies included the following: (1) articles with irrelevant form (case reports/series, review articles, letters to editors, non-English), and (2) studies with irrelevant content (e.g., experimental studies, not related to the effects of melatonin on COVID-19, studies focused on the mechanism, bioinformatics studies). The full texts of the remaining studies were evaluated for final eligibility. The evaluated studies included clinical trials (CTs) and Cohort studies that compared the effect of melatonin in the treatment of COVID-19 with the standard of care therapy in patients older than 18. The full study selection is detailed in the flow diagram (Figure 1).

### 2.4 Risk of bias assessment

We evaluated the risk of bias in each included study using the Newcastle-Ottawa quality assessment scale for cohort studies and the Cochrane risk of bias tool for clinical trials (Higgins et al., 2011; Wells et al., 2000).

Two authors (AF and HHM) independently evaluated the risk of bias in each included study using the Newcastle-Ottawa quality assessment scale for cohort studies and the Cochrane risk of bias tool for clinical trials. The Cochrane tool consists of seven domains to judge the risk of bias: random sequence generation, allocation concealment, selection reporting, blinding of participants and personnel, blinding of outcome assessment, ‌incomplete outcome data, and other bias. For each domain, a judgment of “high risk,” “low risk,” and “unclear” is assigned to the article. Based on this tool, an article is categorized as “good quality” if it receives a judgment of low risk in all domains. An article with a high risk of bias in one domain or an unclear risk in two domains is categorized as “fair quality.” Finally, an article receiving an unclear or high-risk assessment in two or more domains is considered to have poor quality. According to the Newcastle-Ottawa scale, the quality of studies is evaluated based on three domains: selection, comparability, and outcome. Studies can be awarded up to 9 stars depending on how they perform in each domain, and those with a star score ≥6 are considered high-quality studies.

### 2.5 Data extraction and synthesis

Relevant data—including the author's name, country of origin, date of publication, study design, patient characteristics (age, gender, ‌severity, existing comorbidities), melatonin (dosage and duration), and outcomes—were extracted and imported into a pre-defined Excel datasheet. Two independent reviewers performed data screening, and disagreements between the reviewers were resolved through consensus. We included studies on adult patients older than 18 years of age with COVID-19 as confirmed by CT scan or RT-PCR in the analysis.

## 3 Results

### 3.1 Study selection

Following the systematic search, 533 articles were found in four databases (131 in Medline/PubMed, 240 in Scopus, 135 in Web of Science, and 27 in Cochrane); 310 articles remained after removing the duplicates. During the review of titles and abstracts of the studies, 266 articles were excluded based on the criteria mentioned in Figure 1, while 29 articles remained for the full-text screening. A further 21 articles were excluded based on the criteria reflected in Figure 1, leaving eight articles for inclusion in the study. From the time of the systematic search to the submission of the article, a manual search was carried out for newly published articles, and two additional articles were added to our study, bringing the total number of the included studies to ten.

#### 3.1.1 Study characteristics

The ten included articles were classified into two observational retrospective cohorts, five randomized controlled trials (RCTs), and three clinical trials (Table 1). Six of these studies were conducted in Iran, one in the United States, one in Mexico, one in Italy, and one in Iraq. The total number of patients involved was 665. Patients were diagnosed by physicians, and the diagnoses were confirmed by polymerase chain reaction (PCR), chest tomography (CT), or both. Table 2 shows the extracted data, including the population of the studies (case and control groups if available), mean age, sex (number of males and females in each group), the severity of the disease, comorbidities, eligibility and exclusion criteria of the studies, and the interventions (in case, and control groups if available).

**Table 1:** Characteristics of the included studies.

Title	First Author's name	Year of publication	Country	Type of study design
Evaluation of Th1 and Th2 mediated cellular and humoral immunity in patients with COVID-19 following the use of melatonin as an adjunctive treatment	Abdolkarim Hosseini	May-2021	Iran	Cohort (retrospective observational study)
Melatonin is significantly associated with survival of intubated COVID-19 patients	Vijendra Ramlall	October-2021	United States	Cohort (retrospective observational study)
A Pilot Study on Controlling Coronavirus Disease 2019 (COVID-19) Inflammation Using Melatonin Supplement	Zahra Alizadeh	August-2021	Iran	RCT
Efficacy of High Dose Vitamin C, Melatonin and Zinc in Iranian Patients with Acute Respiratory Syndrome due to Coronavirus Infection: A Pilot Randomized Trial	Mahboubeh Darban	December-2020	Iran	RCT
Efficacy of a Low Dose of Melatonin as an Adjunctive Therapy in Hospitalized Patients with COVID-19: A Randomized, Double-blind Clinical Trial	Gholamreza Farnoosh	Jun-2021	Iran	RCT
Melatonin effects on sleep quality and outcomes of COVID-19 patients: An open-label, Randomized, Controlled Trial	Seyed Abbas Mousavi	August-2021	Iran	RCT
Anti-oxidants and pentoxifylline as coadjuvant measures to standard therapy to improve prognosis of patients with pneumonia by COVID-19	Adrián Palacios Chavarría	February-2021	Mexico	Clinical trial
NLRP3 inflammasome activation and oxidative stress status in the mild and moderate SARS-CoV-2 infected patients: impact of melatonin as a medicinal supplement	Hadi Esmaeili Gouvarchin Ghaleh	August-2021	Iran	Clinical trial
The Effect of Melatonin on Thrombosis, Sepsis and Mortality Rate in COVID-19 Patients	Zainab Thanon Hasan	October-2021	Iraq	RCT
Efficacy of Prolonged-Release Melatonin 2 mg (PRM 2 mg) Prescribed for Insomnia in Hospitalized Patients for COVID-19: A Retrospective Observational Study	Carolina Bologna	December-2021	Italy	Clinical trial

Abbreviation: RCT, Randomized controlled trial.

**Table 2:** Detailed characteristics of the studies.

Authors	Population		Age (mean)		Sex		Eligibility	Exclusion	Comorbidities	Severity	Intervention	
	Case	Control	Case	Control	Case	Control					Case (melatonin dosage/duration)	Control
Hosseini et al.	20	20	53	52	12 males 8 females	12 males 8 females	Mild to moderate COVID-19 diagnosed by PCR and CT scan	Pregnancy, organ transplant, neurological diseases, viral diseases (such as hepatitis and HIV), and allergy to melatonin	Diabetes, hypertension, cardiovascular diseases, cancer, rheumatic disease	Mild to moderate	9 mg per day/orally 14 days + Standard medication	Standard medication
Ramlall et al.	112	-	≥ 65	-	-	-	Intubated COVID-19 patients diagnosed by nasopharyngeal real-time PCR test or clinically diagnosed	Intubated patients during a surgical procedure	Diabetes, hypertension, coronary artery disease, myocardial infarction, chronic obstructive lung disease, chronic kidney disease, and respiratory disease	After intubation	-	-
Alizadeh et al.	14	17	37.57	34.53	9 males 5 females	8 males 9 females	Aged 21–60 years, mild to moderate COVID-19, diagnosed by a physician, clinical symptoms, and chest imaging	Diabetes, hypertension, pregnancy or breastfeeding, heart disease, obesity, sleep apnea and seizure, chronic obstructive pulmonary disease (COPD), severe kidney or liver problem, patients who received benzodiazepine, fluvoxamine, or zolpidem drugs which extend QT, allergy to melatonin, and depression	-	Mild to moderate	6 mg per day/orally 14 days + Standard medication	Standard medication
Darban et al.	10	10	-	-	-	-	Aged 18–65 years, diagnosed by real-time PCR, admitted to ICU with PaO2/FiO2 < 200 and SaO2 < 94%,	Not received either remdesivir or tocilizumab, history of nephrolithiasis, allergy to study drugs, pregnancy, hepatic diseases, use of fluvoxamine, sodium oxybate and alcohol, history of copper deficiency, and renal failure	-	Severe	6 mg per day/orally 10 days + Standard medication	Standard medication
Farnoosh et al.	24	20	50.75	52.95	14 males 10 females	12 males 8 females	-	Pregnancy or breastfeeding, neurological diseases, chronic hepatitis, kidney failure, use of anti-oxidants, anti-inflammatory and immunosuppressant drugs, known allergy to melatonin.	Hypertension, diabetes, rheumatic disease, cardiovascular diseases, and cancer.	Mild to moderate	9 mg per day/orally 14 days + Standard medication	Standard medication
Mousavi et al.	48	48	51.06	54.77	25 males 23 females	18 males 30 females	Diagnosed by CT or RT-PCR	Diabetes, hypertension, taking anticoagulants such as warfarin, coagulation disorders, and epilepsy.	Diabetes, asthma, renal failure, cardiovascular disease, hypertension, thalassemia, thyroid disorders, chronic obstructive pulmonary disease.	-	3 mg per day/orally 7–10 days + Standard medication	Standard medication
Chavarría et al.	22	22	-	-	-	-	Aged ≥ 18 years, diagnosed by qRT-PCR	Pregnant or breastfeeding, chronic or recent use of steroids, anti-oxidants, or statins, aged younger than 18 years, refused to be included, or not able to grant an informed consent.	Diabetes, hypertension, dyslipidemia, coronary heart disease, chronic obstructive lung disease, and chronic kidney disease.	Moderate to severe	5 mg per day/orally or naso-enteral tube + Pentoxifylline 5 days	Pentoxifylline, 400 mg per day/orally or naso-enteral tube
Gouvarchin Ghaleh et al.	20	20	-	-	-	-	Aged ≥ 18 years, diagnosed by CT or RT-PCR	Pregnancy, organ transplant, neurological diseases, viral diseases (such as HIV and hepatitis), and allergy to melatonin	-	Mild to moderate	9 mg per day/orally 14 days	-
Hasan et al.	82	76	56.8	55.7	58 males 24 females	56 males 20 females	Aged ≥ 18 or less than 80 years, confirmed severe COVID-19 infection	Pregnancy, aged younger than 18 or older than 80 years, lactating female, autoimmune disease, renal or liver impairment, cancer, terminal medical illness, and allergy to melatonin	Diabetes, hypertension, asthma, and ischemic heart disease	Severe	10 mg per day/orally 14 days + Standard medication	Standard medication
Bologna et al.	40	40	71.6	71.8	23 males 17 females	23 males 17 females	Hospitalized patients in the sub-intensive care unit, diagnosed by PCR, pneumonia confirmed by X-ray, or CT, preserved swallowing function	Patients needed invasive mechanical ventilation, severe renal or liver impairment, comatose patients, severe dementing syndrome, severe heart disease, hypersensitivity to the ingredients, any terminal condition	-	-	prolonged-release melatonin 2 mg	-

Abbreviations: PCR, polymerase chain reaction; CT, chest tomography; PaO2, partial pressure of oxygen; FiO2, fraction inspired oxygen; SaO2, oxygen saturation.

### 3.2 Risk of bias assessment

**Figure 2:**
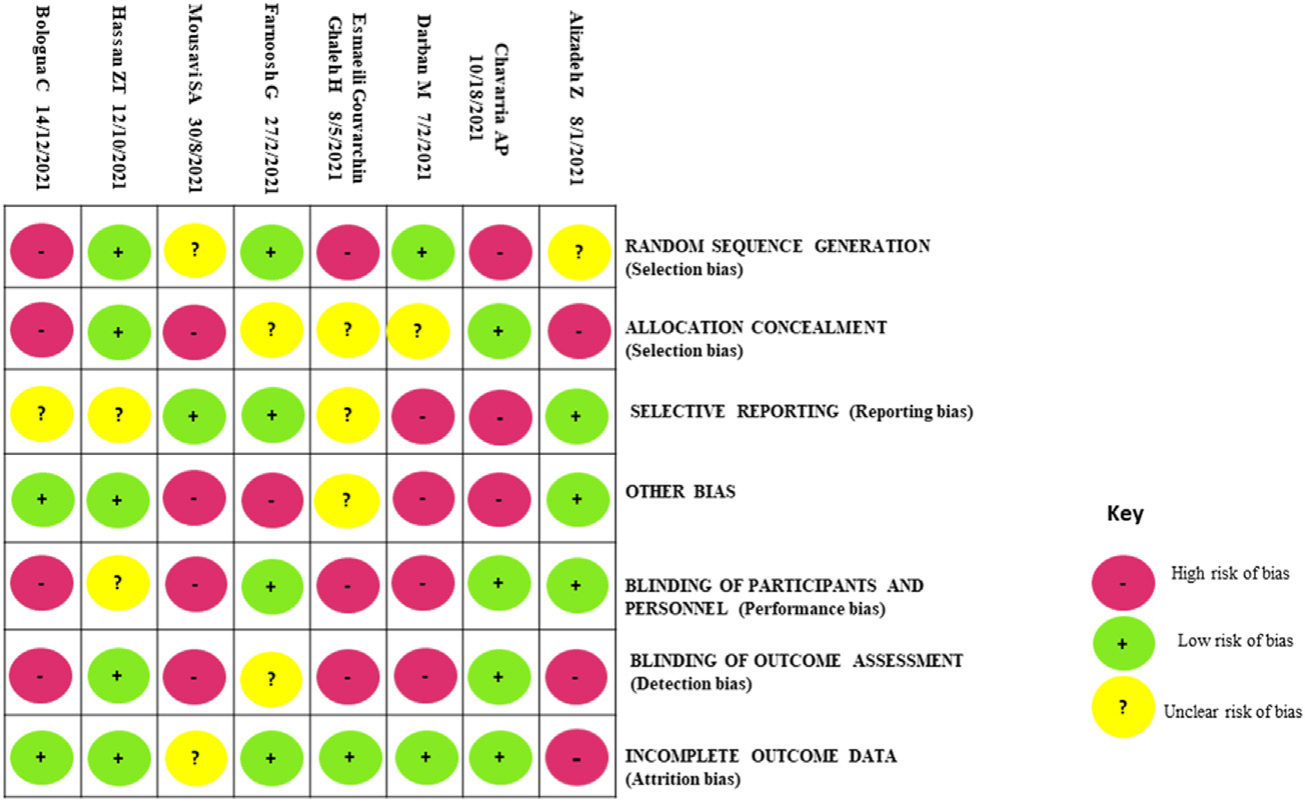
Cochrane risk assessment scale.

The included clinical trials were evaluated for the risk of bias using the Cochrane bias tool. All of the studies, except for the study by Thanon Hasan et al., were determined to have poor quality. Random sequence bias, allocation concealment, selective reporting, blinding of participants and outcome assessors, and other biases received a judgment of “high risk” or “unclear” in most of these studies (Figure 2). Both cohort studies included were of good quality based on the Newcastle-Ottawa scale.

Due to the small number of studies included and the significant percentage of high-risk studies among them, it was not possible to eliminate high-risk studies. This calls attention to the scarcity of high-quality studies in this field.

### 3.3 Effectiveness of melatonin in COVID-19 patients

Here, various effects of melatonin on inflammatory markers, cytokines, gene expression, clinical signs and symptoms, thrombosis, and mortality rate are demonstrated (Table 3).

**Table 3:** Summary of the outcomes.

References	Outcomes	P-value
(Hosseini et al., 2021)	Case group compared to control group:	
	Decreased plasma levels of IL-4	0.037
	Decreased plasma levels of IFN-γ	0.008
	Decreased expression of STAT4	<0.001
	Decreased expression of T-bet	<0.001
	Decreased expression of STAT6	0.024
	Decreased expression of GATA3	0.036
(Ramlall et al., 2020)	Case group compared to non-COVID-19 group:	
	Positive outcome in COVID-19 patients intubation periods requiring mechanical ventilation	0.0000000715
(Alizadeh et al., 2021)	Case group compared to control group:	
	Elevated percentage of recovery	0.057
	Comparison in case group before and after melatonin consumption:	
	Decreased CRP levels	0.005
	Comparison in control group before and after melatonin consumption:	
	Non-significant decrease in CRP levels	0.069
(Darban et al., 2020)	Case group compared to control group:	
	No significant difference in PaO2/FiO2, oxygen saturation, CRP, ESR and LDH levels, and the length of ICU stay	>0.05
(Farnoosh et al., 2021)	Case group compared to control group:	
	Improved clinical signs and symptoms (cough, dyspnea, and fatigue)	<0.05
	Decreased CRP levels	0.045
	Decreased pulmonary involvement	0.045
	Shortened time to hospital discharge	0.021
	Shortened return to baseline health	0.004
(Mousavi et al., 2021)	Case group compared to control group:	
	Improved oxygen saturation	0.003
	Elevated LSEQ score	<0.001
(Chavarría et al., 2021)	Comparison in case group before and after melatonin consumption:	
	Decreased CRP levels	0.004
	Decreased plasma levels of IL-6 in patients with moderate symptoms	0.005
	Decreased procalcitonin levels in patients with moderate symptoms	0.03
	Decreased levels of lipid peroxidation	<0.001
	Elevated levels of nitrites	<0.001
(Esmaeili Gouvarchin Ghaleh et al., 2021)	Case group compared to control group:	
	Decreased plasma levels of IL-1β	0.043
	Decreased plasma levels of TNF-α	0.040
	Decreased plasma levels of malondialdehyde	<0.05
	Decreased plasma levels of nitric oxide	<0.05
	Elevated plasma levels of superoxide dismutase	<0.05
	Decreased expression of ASC	0.037
	Decreased expression of CASP1	0.004
(Hasan et al., 2021)	Case group compared to control group:	
	Decreased thrombosis on day 17	0.037
	Decreased sepsis on day 17	0.000
	Decreased mortality rate	0.000
(C. Bologna et al., 2021)	Case group compared to control group:	
	Increased average total sleep	<0.001
	Reduced episodes of delirium	<0.001
	Reduced length of hospitalization	0.03
	Shortened stay in sub-intensive care unit	<0.001
	Shortened therapy with non-invasive ventilation	<0.001

Abbreviations: IL, interleukin; IFN- γ, interferon γ; STAT, signal transducer and activator of transcription; T-bet, T-box expressed in T cell; GATA, GATA binding protein 3; PaO2, partial pressure of oxygen; FiO2, fraction inspired oxygen; CRP, c-reactive protein; ESR, erythrocyte sedimentation rate; LDH, lactate dehydrogenase; LSEQ, Leeds Sleep Evaluation Questionnaire; TNF, tumor necrosis factor; ASC, apoptosis-associated speck-like protein containing a caspase recruitment domain; CASP1, caspase-1.

Inflammatory markers including c-reactive protein (CRP), erythrocyte sedimentation rate (ESR), and lactate dehydrogenase (LDH), inflammatory cytokines including interleukin (IL)-4, interferon-γ (INF-γ), IL-6, IL-1β, and tumor necrosis factor-α (TNF-α), and expression of some genes including the signal transducer and activator of transcription (STAT)4, STAT6, T-box expressed in T cell (T-bet), GATA binding protein 3 (GATA3), apoptosis-associated speck-like protein containing a caspase recruitment domain (ASC), and caspase1 (CASP1) seem to decrease due to melatonin (Alizadeh et al., 2021; Chavarría et al., 2021; Esmaeili Gouvarchin Esmaeili Gouvarchin Ghaleh et al., 2021; Farnoosh et al., 2021; Hosseini et al., 2021). It is of note that two reports by Hosseini et al. and Esmaeili Gouvarchin Ghaleh et al. are from the same trial of melatonin. In addition, melatonin appears to alleviate some clinical signs and symptoms and accelerate recovery (Alizadeh et al., 2021; Carolina Bologna et al., 2021; Farnoosh et al., 2021; Mousavi et al., 2021; Ramlall et al., 2020). However, there are contradictory results in this regard (Darban et al., 2020). Melatonin use in severe cases reduces thrombosis, sepsis, and mortality rate (Hasan et al., 2021).

## 4 Discussion

Our paper aimed to systematically review eight clinical trial studies and two cohort studies, most of which demonstrate that melatonin supplementation at a dosage of 3–10 mg/day is associated with an improvement in clinical outcomes in COVID-19 patients. Moreover, a retrospective study evaluated the benefits of administering Prolonged-Release melatonin 2 mg in COVID-19 treatment. This study revealed a significant reduction in delirium risk and hospitalization duration, as well as improved quality of sleep (C. Bologna et al., 2021). Although Darban et al. showed that consumption of melatonin in severe COVID-19 does not have a significant effect on inflammatory markers, length of ICU stays, and clinical outcomes (Darban et al., 2020).

Also, Sahu et al. investigated the effect of melatonin dosage (none, 3, 6, or 9 mg) in 706 COVID-19 patients (348 of whom received melatonin) with a mean age of 65.1 predominantly, body mass index >30 (52.1%), male (57.5%). They showed that melatonin (6 or 9 mg/day) is associated with elevated mortality, but this correlation was not statistically significant. In addition, the length of hospital stay was longer for these patients (p < .001) (Sahu et al., 2021).

### 4.1 Effect of melatonin on immune responses in COVID-19 patients

Previous studies have pointed to oxidative stress and inflammation as the two main events implicated in the pathogenesis of viral infections (e.g., influenza, Ebola, Venezuelan equine encephalitis virus, respiratory syncytial virus, hepatitis). Lymphopenia, hyper-inflammatory state, and oxidative stress play principal roles in the pathogenesis of COVID-19 (Bahrampour Juybari et al., 2020). Melatonin has various properties such as immunoregulatory, anti-inflammatory, and anti-oxidant (Zhang et al., 2020). The safety and efficacy of melatonin have been extensively examined in different studies, both in vitro and in vivo, and in a wide range of doses (Colunga Colunga Biancatelli et al., 2020; Huang et al., 2010; Zhang et al., 2020).

Increased expression of immune cell regulatory genes in COVID-19 patients leads to excessive immune response and cytokine storm. Hosseini et al. studied 20 COVID-19 patients who were given a 9 mg/day dose of melatonin and observed decreased gene expression (e.g., T-bet, GATA3, STAT4, STAT6, CAS, CASP1). These genes play a crucial role in immune response regulation (Hosseini et al., 2021).

Our results demonstrate that melatonin consumption for 14 days significantly decreases plasma levels of IL-4, IL-2, IL-1β, IFN-γ, and IL-6 (Esmaeili Gouvarchin Ghaleh et al., 2021; Hosseini et al., 2021) in COVID-19 patients. Therefore, melatonin acts as an immune regulator. In accordance with previous investigations, our results show that melatonin inhibits signaling pathways of the nuclear factor kappa B (NF-κB), which plays an important role in the reduction of inflammatory genes expression. Also, IL-6 is well known as an important biomarker for COVID-19 patients (Carrillo-Vico et al., 2005; Guerrero and Reiter, 2002; Huang et al., 2008; Zhao et al., 2018). However, Sahu et al. revealed that the COVID-19 patients who were given any dose of melatonin did not show any significant alterations in their Lymphocyte count, Ferritin, and CRP (Sahu et al., 2021).

#### 4.1.1 Effect of melatonin on anti-oxidant activity in COVID-19 patients

We found the use of melatonin had a significant impact on reducing oxidant agents (e.g., lipid peroxidation (LPO), nitric oxide, malondialdehyde) and increasing anti-oxidant agents (superoxide dismutase (SOD), nitrites) (Chavarría et al., 2021; Esmaeili Gouvarchin Esmaeili Gouvarchin Ghaleh et al., 2021). Huang et al. showed that melatonin inhibitor affects pulmonary oxidative stress caused by respiratory syncytial virus infection in mice (Huang et al., 2010).

### 4.2 Effect of melatonin on coagulopathy in COVID-19 patients

It has been shown that angiotensin-converting enzyme 2 (ACE-2) functions as the coronavirus's receptor and is widely expressed in the vascular endothelial and alveolar epithelial cells. Inclusion bodies and immune cell recruitment in viral endothelial cells directly induce endothelial dysfunction and cellular apoptosis. Endotheliitis in severe COVID-19 patients can lead to the release of high amounts of inflammatory mediators and the increased formation of neutrophil extracellular traps (NETs), which affect D-dimer levels, which are evident in intravascular thrombosis (Ma et al., 2019; Varga et al., 2020).

Lotufo et al. conducted an in vivo study in which they made a local injection of melatonin and found that reduced endothelial cell interaction with neutrophils leads to a reduction of vascular permeability (Lotufo et al., 2006).

In another study on 46 healthy men, the administration of 3 mg of oral melatonin revealed that plasma melatonin level is inversely correlated with the levels of fibrinogen (p = 0.022) and FVIII: C (P = 0.037) (Wirtz et al., 2008). Hasan et al. treated 82 severe COVID-19 patients with 10 mg of oral melatonin for two weeks. They observed that melatonin could significantly reduce the development of sepsis and thrombosis, thus bringing down the mortality rate in comparison with the control group (Hasan et al., 2021).

So far, three systematic reviews have been published on the association between melatonin and COVID-19. Gholizadeh M et al. conducted a systematic review including four observational studies, two molecular studies, and one animal study. Molecular and cellular studies showed a reduction in viral load and inhibition of proteases of the severe acute respiratory syndrome coronavirus 2 (SARS-COV-2) virus. In a mouse model, reduced acute lung damage was observed. In human studies, reduced lung damage and a shorter ventilation period have been demonstrated (Gholizadeh et al., 2021a). Corrao S et al. performed another systematic study to investigate the effect of melatonin on inflammatory markers, showing that melatonin at a dosage of 5–25 mg/day is effective in decreasing the levels of IL-6, CRP, and TNF (Corrao et al., 2021). Lan et al. carried out a systematic review and meta-analysis on three clinical trials. This study demonstrated that the consumption of melatonin in COVID-19 patients resulted in a higher clinical recovery rate (odds ratio: 3.67; p = 0.02) (Lan et al., 2022).

This systematic review included ten new articles to evaluate the effect of melatonin supplements on patients with COVID-19. Two articles (Esmaeili Gouvarchin Ghaleh et al., 2021; Hosseini et al., 2021) have come from the same study, and there is a concern of duplication, but in the interest of reporting the results completely, we included both of them in this study. Since only four of the included studies reported effect sizes, it was not possible to conduct a persuasive meta-analysis as statistical tests would have insufficient power to detect publication bias due to the small number of studies. Furthermore, the four studies that reported effect sizes used different methods to do so. Two studies calculated the mean, while the other two calculated the median effect size. Therefore, subgroup analysis to deal with possible heterogeneity, sensitivity, or influence analysis would be inconclusive because of the wide confidence intervals of combined effect sizes, resulting from the low number of studies. Therefore, we decided not to conduct a meta-analysis review since combining the findings of these four studies was not logical, given their different types of effect sizes.

**Figure 3:**
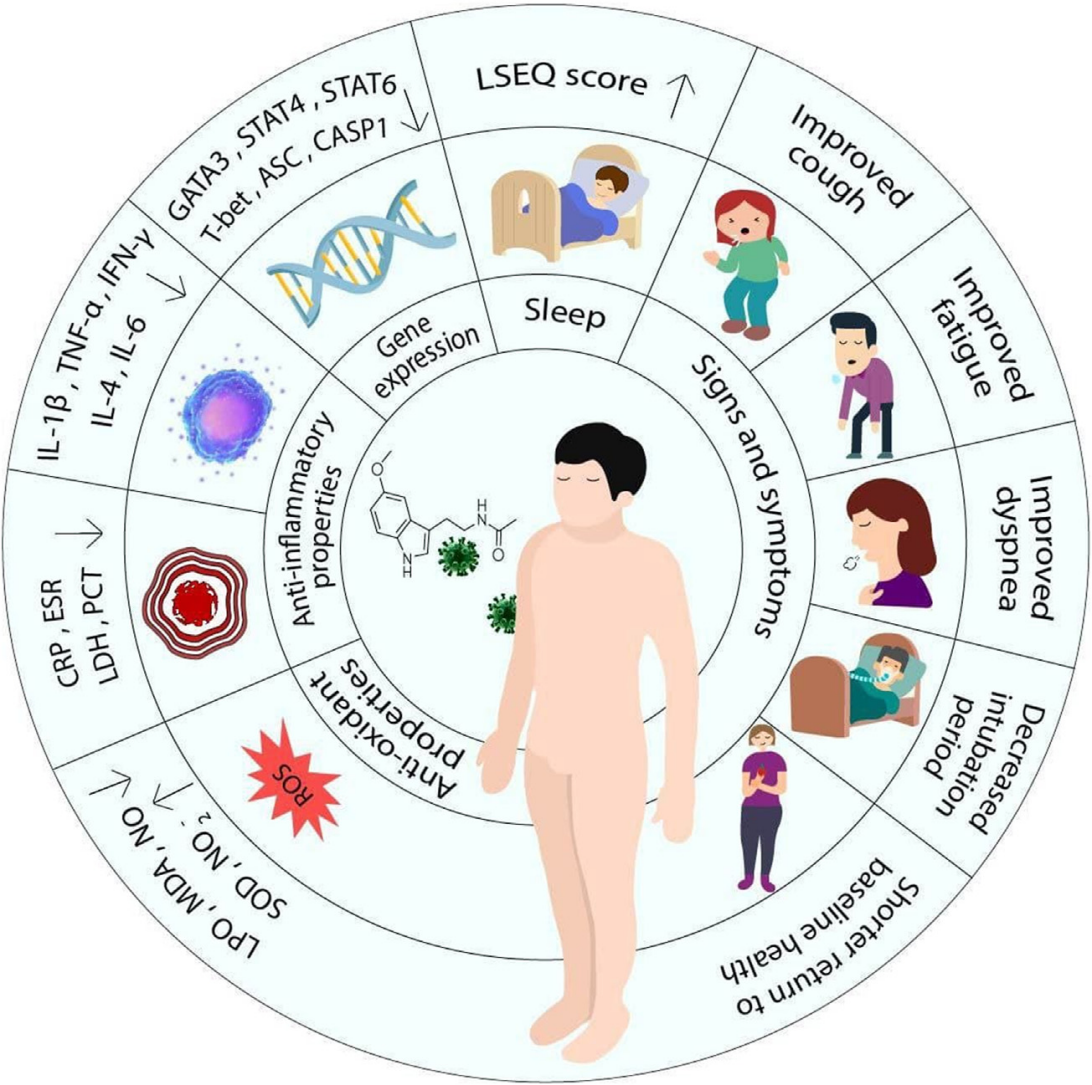
Summary of the effects of melatonin consumption in COVID-19 patients. Abbreviations: ROS, Reactive oxygen species; LPO, lipid peroxidation; SOD, superoxide dismutase; NO, nitric oxide; MDA, malondialdehyde; NO_2_^−^, nitrite; CRP, c-reactive protein; ESR, erythrocyte sedimentation rate; LDH, lactate dehydrogenase; PCT, procalcitonin; TNF, tumor necrosis factor; IL, interleukin; IFN- γ, interferon γ; ASC, apoptosis-associated speck-like protein containing a caspase recruitment domain; CASP1, caspase-1; STAT, signal transducer and activator of transcription; T-bet, T-box expressed in T cell; GATA, GATA binding protein 3; LSEQ, Leeds Sleep Evaluation Questionnaire.

Figure 3 summarizes the positive findings of the studies.

## 5 Conclusion

This systematic review shows that melatonin could be a potential adjuvant in the treatment of COVID-19 patients if administered for two weeks and can possibly help to reduce recovery time, mortality rate, and the likelihood of coagulopathy disorder or sepsis. Furthermore, it can improve patient outcomes during intubation. So far, there have been no high-quality, large-scale studies to establish or reject the effectiveness of melatonin in the treatment of COVID-19, and more high-quality randomized clinical trials are needed to reach a definitive conclusion as to whether melatonin can be a reliable supplemental treatment for the COVID-19 patients.

## Declarations

### Author contribution statement

All authors listed have significantly contributed to the development and the writing of this article.

### Funding statement

This research did not receive any specific grant from funding agencies in the public, commercial, or not-for-profit sectors.

### Data availability statement

Since this study is a systematic review, no original data is available and all the extracted data is in the main manuscript and tables.

### Declaration of interest’s statement

The authors declare no conflict of interest.

### Additional information

Supplementary content related to this article has been published online at [URL].

## Appendix A Supplementary data

PRISMA 2020 Checklist (2) PRISMA 2020 Checklist (2)
